# Outlines of the Arteries, with Short Descriptions

**Published:** 1846-07

**Authors:** 


ART. VIII.—1. *Outlines of the Arteries, with short Descriptions.* By JOHN NEILL, A.M., M.D.—*Philadelphia,* 1845. Royal 8vo.

2. *Outlines of the Nerves, with short Descriptions.* By JOHN NEILL, A.M., M.D., Demonstrator of Anatomy in the University of Pennsylvania, &c. —*Philadelphia,* 1845. Royal 8vo.

THESE two books are abridged descriptions of the vascular and nervous system of the human body, with illustrations, and belong to a class of works that have for their object the making anatomy easy. Now, much as we should desire to see that end attained, we must assure our readers that the royal road to it will not be found in these books. We think the writer must hold that common but erroneous opinion, that an abridgment of the science of anatomy is made by the omission of important facts, and the disregard of common accuracy in statement; so that brevity and generalities are used by the authors referred to as the recipe for the manufacture of the several 'Outlines,' 'Manuals,' 'Guides,' &c. for the use of students and beginners. Fully conscious of the advantage of a good elementary treatise to guide and encourage the learner in his labours, we must protest against such books as these. We generally look suspiciously at first books of young authors, published for the purpose of simplifying difficulties, for such are commonly found to be intended to bring the writers into notice, rather than to assist the inquirer after knowledge. We have examined with some care the books of Dr. Neill, and are forced to state that, as publications, they are neither suitable to the present time, nor calculated to bring honour to the author: they are 'Outlines' made after the worst type.

The pamphlet on the arteries,—it contains but 30 widely-printed pages,—is certainly not fitted to advance the knowledge of the vascular system of the body. The description is loose, unprecise, and often inaccurate, giving in fact little more than what might be inferred from the name of a vessel. All recent observations on the anatomy and surgical anatomy of the vessels are entirely omitted; and the badness of the illustrations exceeds anything we have seen. With one or two exceptions that seem to be copies from dried parts of the body, the figures are merely imaginary, and therefore full of errors; arteries that should be covered by muscles are represented altogether on the surface of the body, as in Plate I; and in many places the descriptions and the drawings do not correspond.

The part containing the nervous system has been cast in the same mould as the other; it contains the same number of pages—almost the same number of lines. If both treatises were not so faulty as to render it difficult to say which is most imperfect, perhaps that on the nerves, though more aspiring in the way of illustrations, might at once be pronounced the more erroneous of the two. Regardless of the progress that has been made in the anatomy of the nervous system within the last few years—we will not say unacquainted with it—the author adopts the old, and now considered erroneous views of the sympathetic and the ganglia in connexion with the fifth cranial nerve. And there is moreover omission of fact and inattention to accuracy all through the book. It would appear as if the writer had taken some old copy of a system of anatomy, and compiled his 'Outlines' from it without discrimination.

